# Chronically Symptomatic Patients with Undetectable Gall Bladder on Ultrasonography Could Benefit from Early Cholecystectomy

**DOI:** 10.1155/2013/630753

**Published:** 2013-01-16

**Authors:** Stephen D. Adams, Simon C. Blackburn, Victoria A. Adewole, Anies A. Mahomed

**Affiliations:** Department of Paediatric Surgery, Royal Alexandra Children's Hospital, Eastern Road, Brighton BN2 5BE, UK

## Abstract

90 percent of symptomatic patients undergoing cholecystectomy have cholelithiasis with 10% categorized as asymptomatic cholecystitis. In both instances, the gallbladder is evident on ultrasonography. In children with symptomatic biliary dyspepsia, the decision to proceed to cholecystectomy is made difficult if choleliths are not seen on ultrasonography. This decision is made even more difficult if the gallbladder itself is not seen on repeated imaging. In a cohort of 54 cholecystectomies, 3 cases, with recurrent right upper quadrant pain and undetectable gallbladders on repeat ultrasonography, were identified. After prolonged observation all underwent successful cholecystectomy. Histology demonstrated a markedly fibrotic and thickened gallbladder in all. Given this experience, we suggest that nonvisibility of the gallbladder, in fact, maybe be a feature of a chronic acalculous cholecystitis. We advise consideration of cholecystectomy for chronic biliary dyspepsia where repeat ultrasonography fails to demonstrate a gallbladder.

## 1. Introduction

Ten percent (2–15%) of all acute cholecystitis is not associated with cholelithiasis [1]. Acute acalculous cholecystitis (AAC) has classically been thought of as a disease of the critically ill patient, usually past their 6th decade and receiving intensive care support for another condition. Increasingly, however, AAC is being recognized in younger patients with no significant comorbidity [2].

Chronic acalculous cholecystitis (CAC), chronic biliary symptoms without radiographic evidence of stones, is also being increasingly diagnosed as a disease entity [3]. The evidence to support cholecystectomy as the treatment of choice for CAC is developing; however, the previously reported short-term benefits may not be reflected to the same degree in longer-term follow-up studies [4]. The imaging findings to indicate AAC are well outlined in the literature [5]. The findings suggesting CAC, on the other hand, are rather more nebulous and it has, therefore, been previously considered a diagnosis of exclusion. 

We have recent experience of 3 cases where CAC has been the final diagnosis, with the repeated abdominal sonographic findings being of a nonvisible gallbladder. We wished to examine this as a possible radiographic feature of CAC.

## 2. Materials and Methods

We maintain a detailed prospective record of all laparoscopic procedures undertaken by a single surgeon in a tertiary paediatric setting. 

A cohort of cholecystectomies undertaken laparoscopically over a 15-year period is reviewed with emphasis on the clinical presentation and ultrasonographic findings. Cases with undetectable gallbladders were studied in more detail.

## 3. Results and Discussion

Fifty-four cases with mean age of 12.32 years (SD 3.82), male : female ratio of 1 : 2, underwent laparoscopic cholecystectomy. 

Median postoperative stay was 1 day (range 0–4 days). There were no conversions to open surgery and mean operating time is recorded as 81 minutes.

Preoperative ultrasonography was performed at least once in all cases. A gallbladder was clearly seen in all but 3 cases with cholelithiasis documented in 46 cases (Table 1).

The 3 cases; 2 females and a male aged 16, 17, and 8 years, respectively, with recurrent RUQ pain had undetectable gallbladders on repeated ultrasonography. The studies were performed in the fasting state, by skilled operators, over at least an 8-month period. These three children were all referred from the medical team after extensive investigation to exclude other causes of their pain, all underwent at least 2 abdominal ultrasound examinations by radiologist experienced in paediatric sonography. After a prolonged observation period, all successfully underwent laparoscopic cholecystectomy.

In terms of the procedures themselves, the operating surgeon subjectively graded the difficulty level in each case as standard, moderately difficult, or difficult. Of the nonvisible gallbladders, 2 were difficult and 1 standard. This is in the context of 31% of the other procedures being recorded as moderately difficult and 20% as difficult. The difficulties recorded were (1) gallbladder stuck to gallbladder bed and (2) foreshortened cystic duct—difficulty with dissection. 

There was one complication recorded in the nonvisible gallbladder group, in a child with previous abdominal operations to place ventriculoperitoneal (VP) shunts; an iatrogenic small bowel perforation was noted and repaired. Discharge home was not delayed beyond 24 hours postoperatively in any of this group and recovery was otherwise uneventful. This child is the only one of this group that complains of any ongoing abdominal pain; however, this is central and functional rather than in the right upper quadrant. Ten percent of the cholelithiasis group had some degree of abdominal pain at follow-up visits.

Histology demonstrated a markedly fibrotic and thickened gallbladder wall in all 3 cases, with microscopic features to support chronic inflammation. The diagnosis of CAC is suggested by these histological features in the excised specimens in the 3 cases of nonvisible gallbladder. 

Previously published reports show a pattern of CAC presenting in otherwise fit children [6], in our small series one patient had treated hydrocephalus. The frequency of CAC as a proportion of all children with cholecystitis is not well defined, but seems to be significantly higher than in adults and may be as high as 30% [6]. Cholecystitis is, however, a relatively uncommon pathology in children; therefore, paediatric CAC is an even rarer phenomenon. 

Biliary dyskinesia (BD) is characterized by symptomatic biliary colic in the absence of gallstones [7]. This description encapsulates the presenting features in our 3 cases. In this situation, therefore, we would propose that BD be considered to as a clinical diagnosis and CAC a histological one. The treatment recommended by many for BD is cholecystectomy and the short-term outcomes are good, although there is some doubt about the longer-term efficacy of this treatment for BD in children [7].

Sonographic findings in CAC are often normal, other imaging modalities that may provide more information include cross-sectional imaging (magnetic resonance (MR), computed tomography (CT)) and scintigraphy or sonography with cholecystokinin (CCK) administration to calculate the gallbladder ejection fraction. These later 2 tests are reported to be the more definitive in diagnosing CAC [8–10]. Cross-sectional imaging, particularly MR, can be difficult to obtain in younger children without general anaesthetic, CT is much quicker but has the dual negatives of less useful information and a relatively high dose of ionizing radiation. The literature discussing imaging in CAC does not seem to touch on the chronically contracted, sonographically nonvisible gallbladder.

One of our patients with previous VP shunts had CAC and underwent a difficult and complicated laparoscopic cholecystectomy. There is no good evidence linking the presence of a VP shunt to CAC; however, the presence of dense upper abdominal adhesions is well reported in these cases and this may go some way to explaining the complex nature of that case [11]. The other two cases in the nonvisible gallbladder group and indeed all other cases in this series had no recorded early complications. 

This study is limited by its size and retrospective nature. The patients were selected from a cohort of children who had undergone cholecystectomy, a small proportion of these children had been noted to have a ultrasonographically nonvisible gallbladder. A formal study examining the potential link between this finding in children with symptomatic gallbladder dyspepsia and the diagnosis of CAC would be difficult to establish prospectively due to the rarity of these circumstances. We must, however, remain circumspect as to the nature of these findings.

This is a small series of paediatric cholecystectomies with an interesting observation; that in 3 cases the gallbladder could not be seen on repeated competent ultrasound examinations in the context of recurrent right upper quadrant abdominal pain. The final diagnosis in these cases was chronic acalculous cholecystitis. Published literature pertaining to imaging CAC does not discuss this. We would like to propose that early consideration is given to performing laparoscopic cholecystectomy when a child presents with intractable gallbladder dyspepsia and a nonvisible gallbladder on ultrasound scan.

## 4. Conclusion

Sonographic nonvisualisation of the gallbladder in patients with intractable gall bladder dyspepsia may suggest the possibility of a chronically scarred organ for which a cholecystectomy is indicated. 

## Figures and Tables

**Table 1:** Categorisation of patients on the basis of pre-op sonographic findings.

Abdominal sonographic findings	Number
Cholelithiasis ± signs of cholecystitis	46
Nonvisible gallbladder	3
Thick-walled gallbladder (no stones seen)	2
Sclerotroaphic gallbladder	1
Pericystic oedema	1
Choledochal diverticulum	1
